# Volcanic Rock Materials for Defluoridation of Water in Fixed-Bed Column Systems

**DOI:** 10.3390/molecules26040977

**Published:** 2021-02-12

**Authors:** Wondwosen Sime Geleta, Esayas Alemayehu, Bernd Lennartz

**Affiliations:** 1 School of Chemical Engineering, Jimma Institute of Technology, Jimma University, P.O. Box 378, Jimma, Oromia, Ethiopia; wondeto@gmail.com; 2 Faculty of Agricultural and Environmental Sciences, University of Rostock, Justus-Von-Liebig-Weg 6, 18059 Rostock, Germany; 3 Faculty of Civil and Environmental Engineering, Jimma Institute of Technology, Jimma University, P.O. Box 378, Jimma, Oromia, Ethiopia; 4 Africa Center of Excellence for Water Management, Addis Ababa University, 1176 Addis Ababa, Ethiopia

**Keywords:** adsorption, breakthrough curve, defluoridation, up-flow mode, volcanic rocks

## Abstract

Consumption of drinking water with a high concentration of fluoride (>1.5 mg/L) causes detrimental health problems and is a challenging issue in various regions around the globe. In this study, a continuous fixed-bed column adsorption system was employed for defluoridation of water using volcanic rocks, virgin pumice (VPum) and virgin scoria (VSco), as adsorbents. The XRD, SEM, FTIR, BET, XRF, ICP-OES, and pH Point of Zero Charges (pH_PZC_) analysis were performed for both adsorbents to elucidate the adsorption mechanisms and the suitability for fluoride removal. The effects of particle size of adsorbents, solution pH, and flow rate on the adsorption performance of the column were assessed at room temperature, constant initial concentration, and bed depth. The maximum removal capacity of 110 mg/kg for VPum and 22 mg/kg for VSco were achieved at particle sizes of 0.075–0.425 mm and <0.075 mm, respectively, at a low solution pH (2.00) and flow rate (1.25 mL/min). The fluoride breakthrough occurred late and the treated water volume was higher at a low pH and flow rate for both adsorbents. The Thomas and Adams–Bohart models were utilized and fitted well with the experimental kinetic data and the entire breakthrough curves for both adsorbents. Overall, the results revealed that the developed column is effective in handling water containing excess fluoride. Additional testing of the adsorbents including regeneration options is, however, required to confirm that the defluoridation of groundwater employing volcanic rocks is a safe and sustainable method.

## 1. Introduction

Credible evidence from scientific literature substantiates both beneficial and detrimental effects of fluoride on human health with only a narrow range between intake associated with these effects [1,2]. Consumptions of fluoride in low concentrations (<1.0 mg/L) is an essential micronutrient for the healthy development of bone and dental enamel [3]; however, it leads to the development of fluorosis if it is consumed beyond the permissible limit (>1.5 mg/L) [4].

In many parts of the world, groundwater sources are the single largest supply of drinking water. For many rift communities, it may be the only economically viable option for drinking water. In the Ethiopian rift valley, about 40% of deep and shallow wells are contaminated with up to 26 mg/L of fluoride [5,6]. The weathering of primary rocks and leaching of fluoride-containing minerals in soils yield fluoride-rich groundwater in the Ethiopian Rift, which is generally associated with a low calcium content and high bicarbonate concentrations [7,8].

Globally, more than 200 million people, including Ethiopia, rely on groundwater with a fluoride concentration above the permissible level [3,4,9]. According to the Central Statistical Agency of Ethiopia report [10], 3.8% of the population is affected by high-level fluoride concentrations (>1.5 mg/L) in groundwater, which is used for drinking purposes. In general, fluorosis turns out to be the most widespread geochemical-based disease in the East African rift, affecting more than 80 million people [11,12,13,14]. Thus, due to the health effect of high fluoride in groundwater, it is essential to reduce excess fluoride concentrations to the allowable limit (<1.5 mg/L).

So far, various technologies such as coagulation/precipitation, electro-coagulation, membrane separations, ion exchange, and adsorption had been attempted for efficient defluoridation of groundwater [15,16,17,18]. Some of the shortfalls of these techniques include expensiveness, fouling issues, regular maintenance, and complicated operational procedures. In comparison to the techniques mentioned above, the adsorption methodology is still one of the most widely applied methods, taking the lead of high removal efficiency, cost-effectiveness, ease of operation, simplicity of design, and availability of large varieties of adsorbents [19,20].

Various adsorbents have been investigated and reported for the removal of excess fluoride from water in an effective manner. Some of the widely employed adsorbents are La (III)-Al (III)-activated carbon modified by chemical route [21], biomaterial functionalized cerium nanocomposite [22], Quaternized Palm Kernel Shell (QPKS) [23], bone char and activated alumina [24], bone char [25], renewable biowaste [26], MgFe_2_O_4_–chitosan–CaAl nanohybrid [27], carbon nanotube composite [15], Neem Oil-Phenolic Resin Treated Bio-sorbent [17], etc. However, many of these suffer from either time-consuming synthesis procedure, high processing costs, availability of raw materials, or short lifespan, which makes them impractical to be applied in the rift valleys that are essentially impacted by high fluoride concentration in water [1]. Consequently, efforts have been made to obtain easily accessible and long-lasting, low-cost, and efficient adsorbents that may be applied for the purification of water in low-income countries such as Ethiopia.

In recent years, volcanic rocks (VPum and VSco) have received significant interest for pollutant removal due to their valuable properties such as high surface area, low-cost, easy accessibility, good mechanical resistance, and availability in large quantities [28]. The source of these rocks is volcanic magma that formed during volcanic eruptions. Pumice (VPum) is a finely porous rock frothy with air bubbles; Scoria (VSco) is a rough rock that seems like furnace slag [28]. VPum is often formed from rhyolite magma [28], it can also develop from trachytic or dacitic magma. Due to its high porosity and low specific gravity, it has been used for water and wastewater treatment processes [29]. VSco is a vesicular pyroclastic rock with basaltic compositions, reddish-brown to black, denser than VPum, somewhat porous with high surface area and strength. Both volcanic rocks are found in abundance in Europe (Italy, etc.), Central America, Southeast Asia (Indonesia, etc.), and East Africa (Ethiopia, Eritrea, etc.) [28,29]. Although several studies have been conducted on the application of volcanic rocks for pollutants-laden wastewaters [28,29,30,31], very little research has been directed to the defluoridation of groundwater using volcanic rocks.

Previously, defluoridation research has been conducted on batch experiments using natural adsorbents [6,32]. The sorption capacity of adsorbents gained from batch equilibrium is valuable in giving basic information about the effectiveness of the adsorbents. Nevertheless, the data obtained from batch studies may not be appropriate for continuous processes where the contact time for the achievement of an equilibrium might be insufficient [33]. Consequently, studies by different authors [34,35,36] reveal that continuous processes mode (fixed-bed column set-up) yields reliable information about the breakthrough time, appropriate adsorption conditions, and the stability of the adsorption performance which can then be used to evaluate the potential of prepared adsorbents for industrial applications [1]. Therefore, there is an interest to conduct adsorption studies in a flow-through system.

The primary objectives of the current work were to (i) investigate the fluoride sorption capacity of VPum and VSco in fixed-bed column set-up, (ii) compare the adsorption properties of both adsorbents with each other, (iii) assess the fluoride adsorption mechanisms with respect to varying solution pH, adsorbent particle size, and flow rate, (iv) deeper analyze the adsorption processes employing mathematical models such as the Adams–Bohart and Thomas model, and (v) finally verify the suitability of the models for the design of flow-through systems for the removal of fluoride from aqueous solutions.

## 2. Results and Discussions

### 2.1. Characterization of Adsorbents

#### 2.1.1. Crystalline Structures and Material Properties and Experimental Conditions

**Figure 1:**
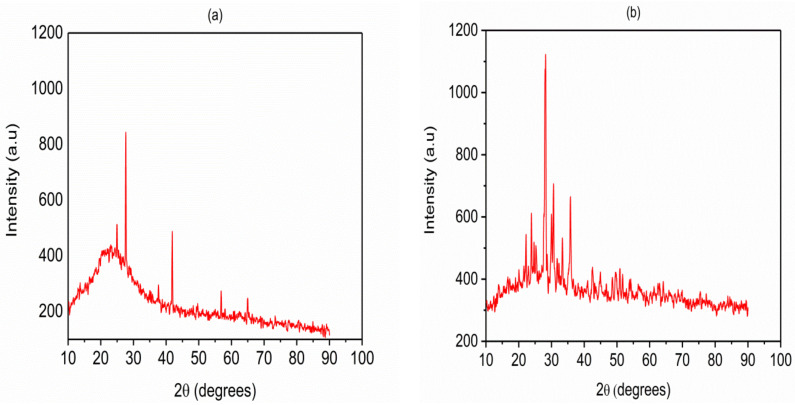
XRD patterns for (**a**) virgin pumice (VPum) and (**b**) virgin scoria (VSco).

The crystalline phases of VPum and VSco were characterized using the X-ray diffraction (XRD) instrumental technique. The mineralogical composition of the adsorbents was characterized by matching the X-ray diffractogram (Figure 1a (VPum), b (VSco)) with the database of the X’pert HighScore Plus software package (Version: 2.2b (2.2.2)). The results showed that the main crystalline phases in VSco were Silicon Oxide (SiO_2_), Albite low (Na(AlSi_3_O_8_)), whereas Hematite (Fe_2_O_3_) and Silicon Oxide (SiO_2_) and Albite high (Na(AlSi_3_O_8_)) are the dominant components of VPum. The presence of crystalline phases in VPum samples can be ascribed to the peaks at 2θ = 24.9°, 27.6°, 27.7°, 37.7°, 41.9°, 58.0°, 64.9°and 65.0°, while that of VSco sample appeared at 2θ = 22.2°, 23.9°, 23.9°, 23.9°, 28.2°, 30.0°, 33.9°, and 35.8°. The detected dome in both samples between 2θ = 10° and 40° is an indication of amorphous material. The amorphous phase(s) present in the adsorbents was estimated by the calibration method. This method makes use of the integrated counts associated with the amorphous and crystalline fraction (Equation (1)) [37].

(1) $$C_{m}\left(\%\right)=\left[\frac{C_{\mathrm{pa}}}{A_{\mathrm{pa}}+C_{\mathrm{pa}}\;}\right]\times 100$$

where C_m_ is the measured crystallinity, C_pa_ and A_pa_ are the integrated peak areas for the crystalline and amorphous components, respectively. The results revealed that the presence of the amorphous phase (s) in VPum and VSco is 89% and 68%, respectively.

The greater fraction of amorphous phase(s) in VPum compared with VSco possibly origins from simultaneous rapid cooling and depressurization of high-temperature volcano lava. The depressurization produces bubbles by lowering the boiling point of the lava. The simultaneous cooling then freezes the bubbles in the matrix of VPum. Due to rapid cooling, crystals do not have enough time to grow. A similar observation has been reported from the XRD analysis of pumice in previous studies [38,39].

Additionally, the results of material properties and experimental conditions were summarized in Table 1 as shown below.

#### 2.1.2. Chemical Composition

The chemical analysis revealed that the major elements in VPum and VSco, as determined by ICP-OES (Table S1), are Si, Al, and Fe. Other elements were present in comparatively smaller quantities or below the detection limit of the instrument. In our previous study [28], the XRF measurement (Table S1) indicated that the oxides of Si, Fe, and Al were the major constituents of both VPum and VSco.

However, the chemical composition of the adsorbents might changes in time due to weathering processes. Consequently, representative samples have to be checked for possible changes induced due to weathering.

#### 2.1.3. Fourier Transform Infrared (FTIR) Analysis

**Figure 2:**
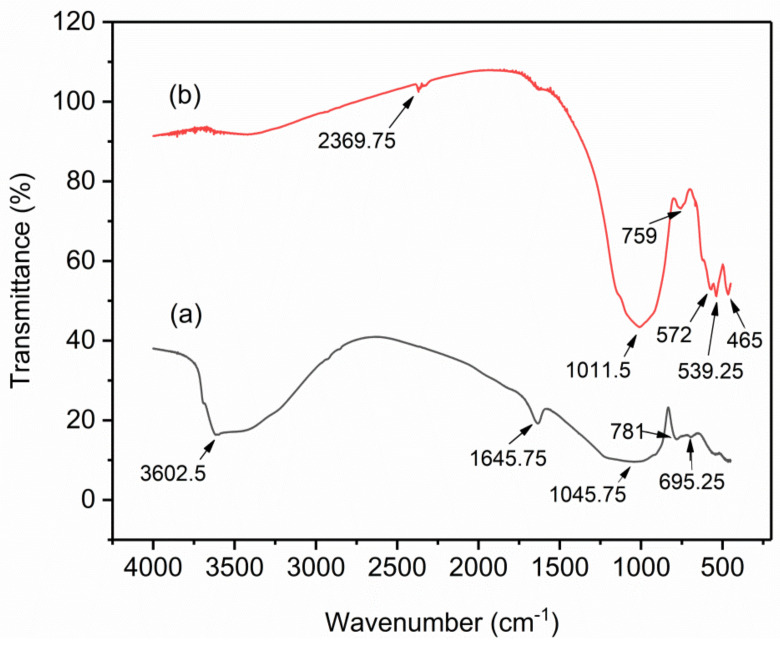
Fourier-transform infrared (FTIR) for (**a**) VPum and (**b**) VSco.

The FTIR spectrums of VPum (Figure 2a) and VSco (Figure 2b) at wavelengths ranging from 4000 to 400 cm^−1^ are shown in Figure 2. Due to the symmetric stretching vibration of Si-O-Si, the absorption band at ~1045.75 cm^−1^ can be assigned to the characteristic peak of (SiO_4_)^2−^ groups in the FTIR spectrum of VPum [39], whereas the band located at ~1011.5 in the FTIR spectrum of VSco can be belongs to the asymmetric stretching vibration of T-O-Si, T = Si or Al [40]. In the FTIR spectrum of VPum, the peaks at ~781 and ~695.25 belong to bending vibrations of Si-O-Si bond [38], whereas the band shown in the FTIR spectrum of VSco at ~759 is related to the stretching vibration of 6-fold coordinated Al(VI)-OH and 6-fold coordinated Al(VI)-O [41]. The small peaks shown in the FTIR spectrum of VSco at ~572 and ~539.25 can be attributed to the symmetric stretching of Si-O-Si and Al-O-Si [40,42], whereas the small band at ~465 belongs to bending vibrations of Si-O-Si and O-Si-O [42]. Certain peaks like the broadening peak at ~3602.5 cm^−1^ in the FTIR spectrum of VPum and sharper peak at ~2369.75 cm^−1^ in the FTIR spectrum of VSco belongs to the asymmetric stretching vibration of -OH bond can be allocated to adsorbed water molecules, whereas the peak at ~1645.75 cm^−1^ in VPum can be allocated to the bending vibration of H-O-H bond [38,39,42]. The most characteristic difference observed between the FTIR spectrum of VPum and FTIR spectra of VSco concerning the band attributed to the asymmetric stretching vibration of -OH bond. This band that is appeared as a broad band at about ~3602.5 cm^−1^ in the FTIR spectrum of VPum becomes sharper and shifts to lower frequencies (~2369.75 cm^−1^) in the FTIR spectrum of VSco indicating that there is a high water content in VPum and could be correlated with less mechanical strength than VSco. Similar observations have been reported for a different system [42].

#### 2.1.4. Scanning Electron Microscope (SEM) Analysis

**Figure 3:**
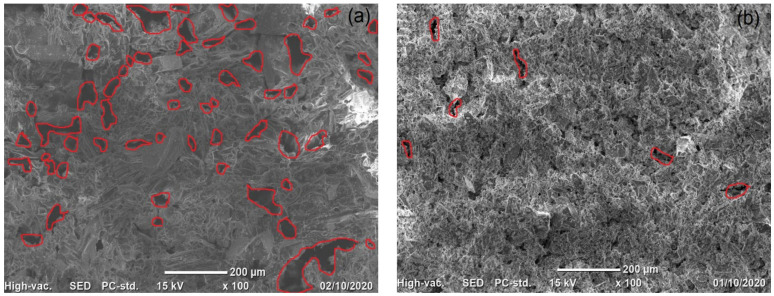
SEM micrographs for (**a**) VPum and (**b**) VSco.

The VPum (Figure 3a) and VSco (Figure 3b) SEM micrographs allowed direct observation of the surface morphology of the adsorbents with a magnification of ×100. The structure of VPum showed that the surface of VPum had an interconnected porous surface [38,43], while VSco had an irregular shape and fibrous cavities (or pores). In addition, it may be said that these pores in VSco were either closed or in open forms (pores) [44]. As seen from the micrographs of the adsorbents, VPum had an interconnected inner porous surface (as indicated in Figure 3a, red-colored), while VSco (Figure 3b) is dominated by the dead-end pores. Consequently, the interconnected internal pore structure in VPum allows for better fluoride accessibility and, hence, better adsorption capacity than VSco.

#### 2.1.5. pH and Point of Zero Charges (pH_PZC_)

**Figure 4:**
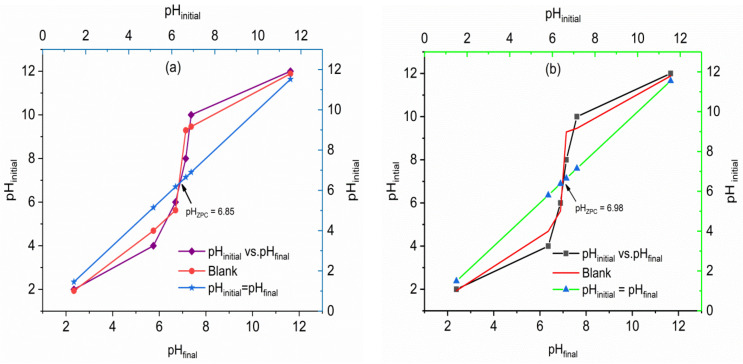
Determination of pH point of zero charges (pH_PzC_) for (**a**) VPum and (**b**) VSco.

The pH of the rock samples in water was found to be 6.65 and 7.20 for VPum and VSco, respectively. The point of zero charges (pH_PZC_) of the adsorbents was identified as 6.85 for VPum and 6.98 for VSco at the intersection of the graph of the initial pH vs. the final pH (Figure 4). The slight difference observed in the adsorbents pH_pzc_ is related to their different characteristics. As can be seen from Table S1, the two volcanic rocks (VPum and VSco) have different chemical compositions, which also influence the surface charge of the adsorbents. This is in agreement with previous studies [30,45], showing the effect of chemical composition on the zeta-potential of different materials. Below these values (pH < 6.85 for VPum and <6.98 for VSco), the surface of the adsorbents is positively charged. Thus, if the pH < pH_PZC_, fluoride could possibly be adsorbed onto the surface of the adsorbents by coulombic attraction [6,46,47]. In addition, the curve for the blank experiment (for blank electrolyte solution 0.01 M NaCl) of both adsorbents is shown in Figure 4. As seen from the blank curve (Figure 4), a pH change without adding the adsorbents was obtained, which confirmed the sorbent dosing is not the only factor to fluctuate the pH of the solution.

### 2.2. Effect of Adsorbents Particle Size

**Figure 5:**
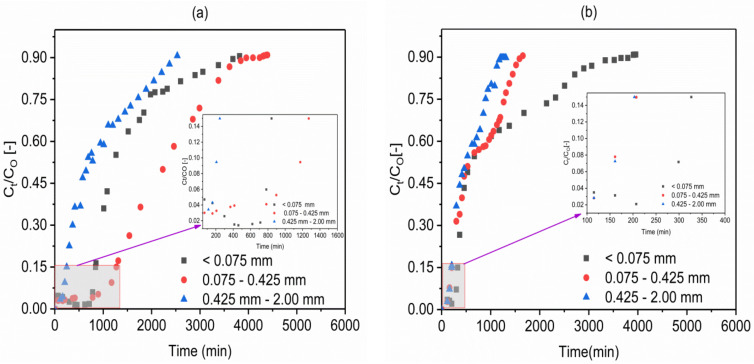
Effect of particle sizes on the breakthrough behavior of fluoride in (**a**) VPum and (**b**) VSco at (pH 2.00; influent fluoride concentration 10 mg/L (C_O_: 10 mg/L); flow rate 1.25 mL/min (Q_O_: 1.25 mL/min; bed depth 10 cm).

The effect of the particles size on the breakthrough behavior of fluoride was investigated for both VSco and VPum with grain size classes of silt to medium sand (<0.075, 0.075–0.425, 0.425–2.00 mm), while maintaining the same initial fluoride concentration (10 mg/L), bed depth (10cm), initial flow rate (1.25 mL/min), as well as solution pH (2.00) (Figure 5a (VPum), b (VSco)). As seen from Figure 5a (VPum) and b (VSco), on reducing the particle size from medium (0.425–2.00 mm) to silt (<0.075 mm) the breakthrough and exhaustion time noticeably increased for VSco, while the breakthrough and exhaustion time was high for VPum at a fine particle size (0.075–0.425 mm). The resulting breakthrough and removal of fluoride parameters are tabulated in Table 2. As can also be seen from Table 2, the amount of total adsorbed fluoride (q_tot_) and the uptake of fluoride was high at silt (<0.075 mm) and fine (0.075–0425 mm) particle size for VSco and VPum, respectively. The smaller particle sizes provide large surface areas and/or sorption sites are more readily available. The results showed that the reduction of particle size of an adsorbent is a significant controlling factor in the fluoride–VSco system (at a particle size of <0.075 mm the fluoride uptake was high). A similar effect was observed for VPum (at a particle size of 0.075–0.425 mm the fluoride sorption capacity was high). However, the effect of particle size on the adsorption capacity is more pronounced for VSco than VPum. That means the pore spaces are more readily available in VPum as compared to VSco, showing that the pore space of VPum is a continuum (skeletal structure) while the pore space of VSco is dominated by dead-end pores. This infers VPum loses its internal porosity at the smallest particle size (<0.075 mm) since the continuum pore space (skeletal structure) is damaged when compared to the fine particle size (0.075–0425 mm) and resulting in smaller internal pore surface areas; consequently, the removal capacity of the adsorbent decreased. On the other hand, the pore space is not readily available in VSco (i.e., the internal pore space of VSco is dominated by dead-end pores). VSco at the smallest particle size (<0.075 mm) is, therefore, expected to have a large surface area, which leads to higher removal capacity compared to the fine particle size (0.075–0425 mm). A similar observation was reported for both adsorbents based on a batch adsorption experiment [28], and a similar remark was also drawn for pumice in the previous study [38]. Moreover, the BET specific surface area (S_BET_) of the adsorbents was determined. As expected, VPum (3.50 m^2^/g) has a larger surface area than VSco (2.49 m^2^/g). Thus, all experiments other than the effect of particle sizes were conducted at a particle size of <0.075 mm for VSco and 0.075–0.425 mm for VPum.

### 2.3. Effect of Influent pH

**Figure 6:**
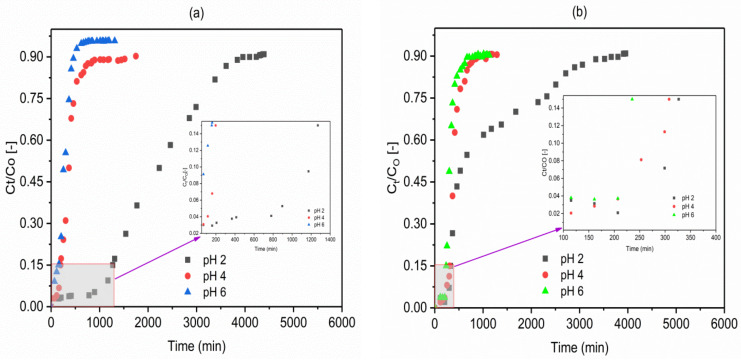
Effect of pH on the breakthrough behavior of fluoride in (**a**) VPum: 0.075–0.425 mm and (**b**) VSco: <0.075 mm (C_O_: 10 mg/L; Q_O_: 1.25 mL/min; bed depth 10 cm).

The influent solution’s pH can noticeably affect the anions sorption on the adsorbents by changing the degree of ionization, the ion speciation, and the adsorbent’s surface charge. Therefore, the effect of solution pH on adsorption of fluoride using VPum and VSco was investigated at different pH (2.00, 4.00, and 6.00) by a separate set of fixed-bed adsorption columns. The breakthrough curves obtained for both adsorbents are shown in Figure 6a,b for a fixed inlet flow rate of 1.25 mL/min, influent fluoride concentration of 10 mg/L, column bed depth of 10 cm, and a particle size of <0.075 mm for VSco and 0.075–0.425 mm for VPum.

As can generally be observed from Figure 6a, b, the adsorption capacity of the adsorbents noticeably increased with decreasing pH. As can also be seen from Table 2 (VPum and VSco), the total amount of fluoride adsorbed (q_tot_) was high for VPum (29.24 mg) and 16.08 mg for VSco at lower pH of 2, and the breakthrough time decreased from 1206 to 135 min for VPum and 415 to 227 min for VSco with an increase in pH from 2 to 6. The volume of water treated at the breakthrough time was higher at pH of 2.00 (1507.5 mL for VPum and 518.03 mL for VSco) than 4.00 (347.5 mL for VPum and 370 mL for VSco) and 6.00 (168.75 mL for VPum and 283.75 mL for VSco). This concludes the occurrence of the breakthrough time was longer, the amount of fluoride adsorbed, and treated water volume was high for a pH of 2.00. As pH varies, surface charge also varies; the sorption of charged species is affected. Therefore, the performance of adsorbents for better adsorption at low pH may be the result of the presence of a large number of H^+^ ions at low pH values, and hence neutralize the negatively charged adsorbent surface [48], consequently dropping the interference of the adsorption of fluoride. In addition, this reality can be elucidated based on the pH value at the point of zero charges of the adsorbents (pH_PZC_ = 6.85 (VPum) and 6.98 (VSco)).

Moreover, the decrease in the adsorption of fluoride at pH 4.00 and 6.00 could also be due to the decrease in the number of H^+^ or electrostatic repulsion of fluoride by negatively charged adsorbent surface [47,49].

Hence, the sorption of fluoride ions is due to an electrostatic phenomenon and surface complexation that perform independently or together for the adsorption of fluoride ions on the adsorbents. The removal mechanism at pH < pH_PZC_ is presumably due to columbic attraction of fluoride by positive surface charges (Equation (2)) and/or ligand exchange reactions of fluoride with surface hydroxyl groups (Equation (3)).

(2) $${\mathrm{MOH}}_{2}^{+}+F^{-}\;↔{\;\mathrm{MOH}}_{2}^{+}---F^{-}$$

(3) $${\mathrm{MOH}}_{2}^{+}+{\;F}^{-}\;↔\;\mathrm{MF}+H_{2}O$$

where, M represents Fe, Al, Si, Ca, Mg, etc.

In this study, an increment in the final pH (pH ~7.20) was observed after the completion of the adsorption experiment, which is consistent with the columbic or ligand exchange type adsorption mechanism shown in Equations (2) and (3) [47]. This can be further explained by the capacity of the adsorbents to maintain a neutral pH after adsorption [6,50]. The capacity to maintain neutral effluent solution pH could be from the amphoteric nature of oxides in both adsorbents (Al_2_O_3_, Fe_2_O_3_, TiO_2_, etc.) when compared with the effect of basic metallic oxides (Table S1). This type of observations were reported for the removal of pollutant in a previous study [6]. Furthermore, the elemental compositions of exchangeable cations also play a critical role in fluoride uptake during defluoridation [51]. There might be a slight increase in electro conductivities of the final solutions, which might not influence the adsorption process [52]. However, additional testing of the effluent solution for various compounds may be required to draw definite conclusions.

It is noted that the effect of pH on the adsorption capacity may be due to the shared impact of pH on the nature of the adsorbent surface, the existence of the adsorbed pollutant (fluoride ion), and the added acid and base to the working solution to adjust its pH. In this study, the optimum and effective removal of fluoride takes place at a pH of 2.00; hence, all experiments other than the effect of pH were conducted at a pH of 2.00 for both adsorbents.

### 2.4. Effect of Flow Rate

**Figure 7:**
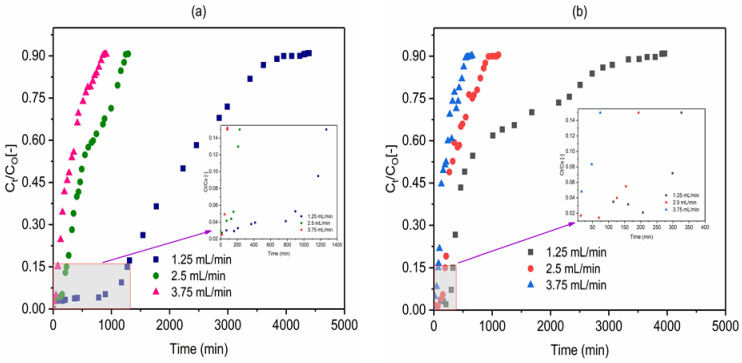
Effect of influent flow rate on the breakthrough behavior of fluoride in (**a**) VPum: 0.075–0.425 mm and (**b**) VSco: <0.075 mm (pH 2; C_O_: 10 mg/L; bed depth 10 cm).

The effect of flow rate on the adsorption of fluoride using VPum and VSco was examined at the flow rates of 1.25, 2.50, and 3.75 mL/min whereas the bed depth (10 cm), influent solution pH (2.00), initial fluoride concentration (10 mg/L), and adsorbents particle size (<0.075 mm (Vsco) and 0.075–0.425 mm (VPum)) were held constant. As indicated in Figure 7a (VPum) and b (VSco), the breakthrough curves become steeper and shifted to the origin with an increasing flow rate while the breakthrough time decreased. The use of a high flow rate decreases the contact time of fluoride in the solution with the adsorbents, thereby allowing earlier breakthroughs to occur. Additionally, increasing the flow rate from 1.25 to 3.75 mL/min decreased the volume of water treated from 1507.5 to 282.69 mL and 518.03 to 256.03 mL for VPum and VSco, respectively (Table 2).

This was further supported by Mass Transfer Zone (MTZ) (Table 2) which increased with increasing flow rate. The total fluoride adsorbed (q_tot_) increased from 4.49 mg to 29.24 mg for VPum and from 3.10 mg to 16.08 mg for VSco (Table 2) as the flow rate decrease from 3.75 to 1.25 mL/min. This results in the increase of the adsorption performance of the column from 17 to 110 mg/kg and 4.2 to 22 mg/kg for VPum and VSco, respectively (Table 2). The increase in sorption efficiency at a low flow rate shows that the adsorbates have sufficient time to penetrate and diffuse deeply into the pores of the adsorbents; hence, intraparticle mass transfer controls the sorption process. This was also verified by MTZ (Table 2) or unused bed, which decreased with decreasing flow rate. In general, at the lower flow rate, the contact time between the adsorbent and the fluoride was higher, resulting in an increased breakthrough time and treated water volume for the continuous column adsorption system. A similar type of observation was reported by various authors for fixed-bed column systems [53,54,55]. In this study, the optimum and effective removal of fluoride takes place at a flow rate of 1.25 mL/min; so, all experiments other than the effect of flow rate were performed at a flow rate of 1.25 mL/min.

### 2.5. Application of the Thomas Model

The values of the Thomas model parameters, K_T_ and q_o_ for both adsorbents shown in Table 3 for different experimental parameters were found from the non-linear optimization techniques according to Equation (18). The non-linear plots of the experimental (designated as exp.) and simulated (designated as cal.) breakthrough curves based on the Thomas model for VPum (a) and VSco (b) at different particle sizes (Figure S1), influent pH (Figure S2), and influent volumetric flow rate (Figure S3) were provided in Supplementary Materials. The results of K_T_, q_o,_ and correlation coefficient (R^2^) are shown in Table 3 for VPum and VSco. From the results, it can be seen that the values of R^2^ range from 0.897 to 0.993 for VPum and 0.901 to 0.973 for VSco.

The high values of R^2^ indicate there were no significant disparities between the experimental data points and calculated data by the Thomas model for all particle sizes, influent solution pH, and influent volumetric flow rate. The observed differences between the experimental data and calculated data from the Thomas model may be due to the characteristic attribute weakness in the model. The Thomas model does not consider the external (film) and intra-particle diffusions in the adsorption system and, therefore, proposes adsorbate–adsorbent surface reactions to control the adsorption rate, hence the breakthrough. However, nearly all adsorption operations are typically not limited to surface reaction kinetics, but are also controlled by external and/or intra-particle diffusion [56,57]. Thus, the perceived disparities in this study indicate external and/or intra-particle mass transfer may be the rate-controlling steps in fluoride adsorption in a fixed-bed column onto the adsorbents. Similar observations were drawn with the kinetic study of fluoride under fixed-bed conditions onto modified pumice [57]. As shown in Table 3, the value of the Thomas rate constant (K_T_) increased as the influent flow rate increased but the value of the maximum solid-phase concentration (q_o_) decreased. A related type of investigation on Thomas constants for different systems was reported by various authors [58,59].

### 2.6. Application of the Adams–Bohart Model

The parameter values of the Adams–Bohart model, K_AB_ and N_O_, as depicted in Table 4 for Vpum and Vsco were similarly determined by non-linear regression analysis according to Equation (19).

From the results presented in Table 4, it can be realized that the values of R^2^ range from 0.911 to 0.993 for Vpum and 0.886 to 0.969 for VSco. The high values of R^2^ designate the applicability of the Adams–Bohart model for describing the entire sorption mechanisms of fluoride onto VPum and VSco under a continuous fixed-bed flow process.

In a similar fashion with the Thomas model, the comparison of the non-linear plots of the experimental and calculated breakthrough curve, based on the Adams–Bohart model, are generally in good agreement for VPum (a) and VSco (b) at different particle sizes (Figure S4), influent solution pH (Figure S5), and influent flow rate (Figure S6) respectively. Only minor disparities were noticed at lower pH (2.00) and particle sizes of 0.075–0.425 mm and <0.075 mm for VPum and VSco, respectively. As seen in Table 4, the values of the kinetic constants were affected by the influent flow rate and increased with increasing flow rate. This presented that external mass transfer in the entire fluoride adsorption mechanisms in the fixed-bed column dominates the overall system kinetics [57,60]. In general, both the Adams–Bohart and the Thomas models could predict very well the entire region of the breakthrough curves for the fluoride-VSco and fluoride-VPum systems. In addition, both the Adams–Bohart model (Equation (19)) and the Thomas model (Equation (18)) are mathematically the same and, therefore, gave similar fit quality.

### 2.7. Comparison of Different Adsorbents on Fluoride Removal

A comparison has been made between volcanic rocks (VPum and VSco) used in this study and previously reported adsorbents for fluoride removal in a fixed-bed column system. The results for some adsorbents are presented in Table 5.

As can be seen from these results in Table 5, the natural VPum used is comparable to cement paste and aluminum modified iron oxide in terms of defluoridation capacity. Values of adsorption capacity per unit surface area are, however, higher for VPum than those of acid-treated bentonite (GHB), kanuma mud, and activated alumina; and higher for VSco than acid-treated bentonite (GHB) and activated alumina (Grade OA-25) (Table 5). Both adsorbents are available in abundance in all parts of the world and are readily available in approximately 1/3 of Ethiopia’s total area and are, hence, favored adsorption materials because of very low supply costs. The adsorbents used are primarily part of the natural environment. However, to improve the specific surface area and hence the defluoridation capacity, surface modification of the natural volcanic rocks may be appropriate.

## 3. Materials and Methods

### 3.1. Materials

In this study, rock samples were collected from volcanic cones (VPum: 8°10′ N 39°50′ E; VSco: 8°33′ N 39°16′ E) of the Main Rift Valley area of Oromia Regional State, East Showa Zone, Ethiopia, around 50–100 km East of Addis Ababa. The rocks are readily available in approximately 1/3 of the country’s total area and are, thus, a preferred adsorption material because of very low supply costs [29,48,66].

### 3.2. Preparations of Adsorbents

The rock samples (VPum and VSco) were washed repeatedly with deionized water until all water-soluble compounds and dust were removed, and thereafter dried at 55 °C for 48 h [30,67]. After cooling samples down to room temperature, they were crushed with a mortar and sieved using different mesh sizes: silt (<0.075 mm), fine sand (0.075–0.425 mm), and medium sand (0.425–2.00 mm) [28,68]. All prepared samples were packed in air-tight plastic bags and stored at a cool and dry place for further use.

### 3.3. Preparations of Adsorbate

All glassware and bottles were thoroughly washed and rinsed with deionized water before usage. Chemicals used were analytical grade reagents and a fluoride stock solution (1000 mg/L) was prepared freshly by dissolving 2.21 g of anhydrous NaF (Merck KGaA, Darmstadt, Germany) in 1000 mL of deionized water. The synthetic fluoride solution of desired concentrations was made by diluting the stock solution. 0.1 M of NaOH and/or 0.1 M HCl solutions were used to adjust the pH values of the fluoride solution utilized in the column experimental experiments.

### 3.4. Adsorbent Characterizations

#### 3.4.1. Crystalline Structures

The crystalline structures of the adsorbents were analyzed by an X-ray diffractometer (XRD-7000, Drawell, Shanghai, China) with Cu Kα as a radiation source (1.54056 Å) generated at 30 kV and 25 mA instrument. The diffractograms were gained with a step width of 2θ and a scan rate of 0.04°/min.

#### 3.4.2. Chemical Composition

The elemental composition of the adsorbents was analyzed using inductively coupled plasma-optical emission spectroscopy (ICP-OES). X-ray fluorescence (XRF) spectroscopy was used to obtain information on the oxide contents of the adsorbents.

#### 3.4.3. Fourier Transform Infrared (FTIR) Analysis

FTIR spectra of the samples were run on KBr pellets. The spectra were recorded over a range of 5000 to 400 cm^−1^ at a resolution of 0.1 cm^−1^ in a PerkinElmer spectrometer (UNSW Sydney, Australia) using a lithium tantalite (LiTaO_3_) detector.

#### 3.4.4. Scanning Electron Microscope (SEM) Analysis

A scanning electron microscope (SEM) (JCM-6000plus, Version 0.2, Peabody, MA, USA), operated at 15 kV, was used to determine the morphologies of VPum and VSco. The characteristics of the adsorbents were compared.

#### 3.4.5. Determination of pH and Point of Zero Charges (pH_PZC_)

The pH of the adsorbents was determined using a pH meter in a 1:10 adsorbent/water ratio as per the standard method [6]. The pH at the point of zero charges (pH_PZC_) of the adsorbents was examined based on the standard method. For this effect, 250 mL of 0.01 M NaCl solution as an electrolyte was positioned in a vessel, thermostated at 298 K, and N_2_ was bubbled through the solution to stabilize the pH by preventing the dissolving of CO_2_ from the air. In 6 Erlenmeyer flasks, 25 mL of the electrolyte was introduced and the pH was adjusted to the required value (2.00, 4.00, 6.00, 8.00, 10.00, and 12.00) by adding 0.1 M NaOH or 0.1 M HCl. The same procedure and method were performed for blank electrolyte solution (0.01 M NaCl). In each beaker, 0.25 g of the rock samples were added and shaken for 48 h. The suspension was subsequently filtrated and the final pH was determined. The point of zero charges (pH_PZC_) was found at the intersection point by plotting the initial pH versus the final pH.

#### 3.4.6. BET Analysis

The specific surface area (*S_BET_*) of the adsorbents was measured using a nitrogen gas adsorption-desorption technique at 77k using surface analyzer equipment (Micrometrics/Gemini-2372). The samples were degassed at 300 °C under vacuum for at least 6 h before analysis. The Brunauer-Emmett-Teller (BET) equation was used to obtain a specific surface area (*S_BET_*). The *S_BET_* values of the two adsorbents (VPum and VSco) are compared.

### 3.5. Column Adsorption Experimental Set-Up and Procedures

Continuous fixed-bed column adsorption studies were carried out to assess the dynamic behavior of fluoride removal by using VPum and VSco. Continuous flow adsorption experiments were conducted in a small-scale cylindrical column of 8.1 cm internal diameter and 10 cm height with an empty bed volume of 515 cm^3^. The column was filled with a weighted amount of adsorbent of different particle sizes (silt: <0.075 mm, fine sand: 0.075–0.425 mm, and medium sand: 0.425–2.00 mm). The same particle size was used if controlling parameters such as pH and flow rate were tested. The bed was conditioned with deionized water (pH: 7.00–7.30) for 12 h (overnight) to ensure a closely packed adsorbent and to avoid the potential occurrence of voids, channeling, or cracking, which can significantly affect the performance of the column.

A synthetic fluoride solution with a concentration of 10 mg/L was pumped to a packed bed column in up-flow mode to avoid channeling caused by gravity. The influent volumetric flow rate varied between experiments but was held constant in a given experiment using an adjustable peristaltic pump (MS-REGLO, Labortechnik-Analytic, Zürich, Switzerland). The experiments were conducted at room temperature (25 ± 2 °C). The effluent column sample was collected using an automatic fraction collector (RFI, MA-RON GmbH, Germany). The constant flow rate was verified by collecting and quantifying the effluent solution at regular time intervals. The column operation was stopped when the concentrations of the fluoride in the effluent exceeded 90% of its initial concentrations. Ion chromatography (930 Compact IC Flex, Metrohm, Switzerland) was used to quantify fluoride concentrations. The instrument uses 3.2 mmol/L Na_2_CO_3_/1.0 mmol/L NaHCO_3_ as eluent, Metrosep A Supp 5–150/4.0 column, and a standard conductivity detector to measure the conductivity of the effluent solutions. The fluoride concentration was measured in the calibration range of 0.2–200 mg/L, contains inline dilution, inline dialysis, eluent degasser, CO_2_ suppressor, and chemical suppressor. Suppression in IC maximizes the detection sensitivity of fluoride ions while reducing the background conductivity of the eluent.

The maximum tolerable breakthrough concentration (C_b_) was 1.5 mg/L (15% of the influent initial concentration of 10 mg/L), which is recommended by WHO [4] as a maximum acceptable level for drinking water.

The effect of experimental parameters such as particles size (silt: <0.075 mm, fine sand: 0.075–0.425 mm, and medium sand: 0.075–0.425 mm), influent solution pH (2.00, 4.00, and 6.00), and influent volumetric flow rate (1.25, 2.50, and 3.75 mL/min) on breakthrough behavior and amount of fluoride removed were examined.

### 3.6. Modeling and Analysis of Fixed-Bed Column Data

A fixed-bed column adsorption performance is well described through the breakthrough curve concept [53]. The time of solute breakthrough and the shape of the breakthrough curve are important indicators for the operational adsorption processes; the breakthrough curve is directly linked to the viability and economics of the adsorption process [54,69]. The breakthrough patterns and according parameters are dependent on the operating conditions of the fixed-bed column such as adsorbent particle size, influent flow rate, and pH of the influent solution. Nevertheless, the pH value may not influence the breakthrough curve in a situation such as when using strongly basic anion exchangers. The primary and significant attribute is the sorbent selectivity to the pollutant, as well as the dynamic exchange capacity and full dynamic capacity of the column [70]. To investigate the performance of the column and to scale-up, the determination of breakthrough parameters is crucial. The breakthrough curves expressed in terms of the ratio of effluent to influent adsorbate concentration (C_t_/C_o_) as a function of time or effluent volume for a given height of the bed reflects the absorbed fluoride from the solution. Time equivalent to stoichiometric capacity (exhaustion time) and time equivalent to usable capacity (breakthrough time) is shown in Equations (4) and (5), respectively [54,59].

(4) $$t_{e}=\int_{t=0}^{t=t_{\mathrm{total}}}\left(1-\frac{C_{t}}{C_{o}}\right)\mathrm{dt}$$

(5) $$t_{b\;}=\int_{t=0}^{t_{b}}\left(1-\frac{C_{b}}{C_{o}}\right){\mathrm{dt}}^{\;}$$

where t_e_ is exhaustion time (min), t_b_ is the breakthrough time (min) at which C_t_ = C_b_ (mg/L) (for the present system, C_b_ = 1.5 mg/L).

The total value of fluoride adsorbed (q_total_: mg) from the column for a given feed concentration and the flow rate was obtained from the area (A) under the breakthrough curve by integrating the adsorbed fluoride concentration C_ad_ (C_ad_ = C_o_−C_t_) (mgL^−1^) versus t (min) and can be obtained from Equation (6) [55,71].

(6) $$q_{\mathrm{total}\;=\;}\frac{\mathrm{QA}}{1000}=\frac{Q}{1000}\int_{t=0}^{t=t_{\mathrm{total}}}C_{\mathrm{ad}}{\mathrm{dt}}^{\;}$$

where t_total_, and Q are the total flow time until saturation of the bed (min), and volumetric flow rate (mL/min), respectively.

Equilibrium fluoride uptake (q_e:_ mg kg^−1^) (maximum capacity of the column) in the column is calculated by Equation (7) as the total amount of fluoride adsorbed (q_total_) per kilogram of dry adsorbent mass (m) at the end of the total flow time [71].

(7) $$q_{\mathrm{eq}\;=\;}\frac{q_{\mathrm{total}}}{m}$$

The effluent volume (V_e_) and treated effluent volume or breakthrough volume (V_b_) of solution can be found from Equations (8) and (9), respectively.

(8) $$V_{e}={\mathrm{Qt}}_{e}$$

(9) $$V_{b}={\mathrm{Qt}}_{b}$$

where, V_e_ is the total effluent volume at exhaustion time (mL), V_b_, total effluent volume at the breakthrough time (mL), Q is the volumetric flow rate (mL/min), t_e_ and t_b_ are exhaustion and breakthrough time (min), respectively.

The Mass Transfer Zone (MTZ) or unused bed length (H_UNB_) can be obtained from Equation (10) [54,59].

(10) $$\mathrm{MTZ}=H_{T}\left(\frac{t_{e}-t_{b}}{t_{e}}\right)$$

where H_T_ is total bed height (cm), t_e_ (min) is exhaustion time, and t_b_ is breakthrough time (min).

The Empty Bed Contact Time (EBCT), which measures the critical depth and the contact time between the solid phase adsorbent and the liquid phase, can be obtained from Equation (11).

(11) $$\mathrm{EBCT}=\frac{V_{B}}{Q}$$

where V_B_ is the volume of a fixed bed (mL) and Q is the flow rate (mL/min).

The bulk density (ρ_b_: gm.cm^−3^), which measures the adsorbent compaction status, and the particle density (ρ_p_: gm.cm^−3^) of the adsorbent can be obtained from Equations (12) and (13), respectively [72].

(12) $$\rho_{b}=\frac{m_{\mathrm{ads}}}{V_{t}}$$

(13) $$\rho_{p}=\frac{m_{\mathrm{ads}}}{V_{t}-\;V_{v}}$$

where m_ads_ is the dry mass of adsorbent (mg), and V_t_ is the bulk volume (cm^3^) which includes the volume of adsorbent (V_B_:cm^3^) and the pore space between the adsorbent particles or void volume (V_v_:cm^3^).

The void volume (V_v_:cm^3^) of the adsorbent can be found from Equation (14) [72].

(14) $$V_{v}\;=\frac{W_{\mathrm{Sat}}-W_{\mathrm{dry}}}{\rho_{w}}$$

where W_dry_ is the weight of dry adsorbent (g), W_sat_ is the weight of saturated adsorbent (g), and $\rho_{w}$ is the density of water (g cm^−3^).

The total porosity of the adsorbent (ε_b_) can be obtained from Equation (15) [72].

(15) $$\varepsilon_{b}\;=1-\frac{\rho_{b}}{\rho_{p}}$$

The filter (superficial) velocity (V_f_:cm min^−1^) and effective (interstitial) velocity (V_I_:cm min^−1^) can be found from Equations (16) and (17), respectively [72].

(16) $$V_{f}=\frac{Q}{A}$$

(17) $$V_{I}=\frac{Q}{A\times \varepsilon_{b}}$$

where A is the cross-sectional area of the fixed-bed (cm^2^) and Q is the flow rate (cm^3^min^−1^).

### 3.7. Fixed–Bed Column Breakthrough Curve Modeling

The successive operation of a small scale column towards industrial applications can be well elucidated with the help of some models. Various models have been reported for predicting the breakthrough performance in fixed-bed adsorption [57,73]. In this study, the two most important and widely used mathematical models, the Thomas model and Adams–Bohart model, have been applied to the column experimental data for describing the dynamic behavior of fluoride adsorption using VPum and VSco in a fixed-bed column filter.

#### 3.7.1. Thomas Model

The Thomas model [74] is one of the most extensively employed kinetic models to predict fixed-bed column performance. In addition to the prediction of the breakthrough curve for the effluent, the model can be used to determine the maximum uptake of adsorbate and adsorption rate constant [74]. The non-linear form of the Thomas model can be described as follows Equation (18), [75].

(18) $$\frac{C_{t}}{C_{o}}=\frac{1}{1+\exp\left[K_{T}q_{o}\frac{m}{Q}-K_{T}C_{o}t\right]}$$

where C_o_ (mg/L) is the initial solute concentration, C_t_ (mg/L) is the solute concentration at the time, t, Q (L/min) is the volumetric flow rate, q_o_ (mg/kg) is the maximum solid-phase concentration of solute (maximum column adsorption capacity), K_T_ is the Thomas rate constant (L/min mg), and m (kg) is the packed dry mass of the adsorbent in a fixed-bed.

#### 3.7.2. Adams–Bohart Model

The Adams–Bohart model [76] was developed for the analysis of the dynamics of fixed-bed based on the assumption that the adsorption rate is proportional to both the residual adsorbent and adsorbate concentration. The nonlinear form of the Adams–Bohart model (Equation (19)) [77], was used for the prediction of breakthrough curves and model parameters.

(19) $$\frac{C_{t}}{C_{o}}=\frac{1}{1+\exp\left[K_{\mathrm{AB}}N_{o}\frac{Z}{v}-K_{\mathrm{AB}}C_{o}t\;\right]}$$

Where K_AB_ (L/mg min) is the kinetic constant, *v* (mL/min) is the linear flow rate, Z (cm) is a column bed depth, and N_O_ (mg/L) is the saturation concentration (adsorption capacity of the adsorbent per unit volume of the bed), and time t (min) ranges from the start to fluoride breakthrough point. The linear flow rate was determined by Equation (20).

(20) $$v=\frac{Q}{A}$$

where Q (cm^3^/min) is the volumetric flow rate, and A (cm^2^) is the cross-sectional area of the bed [60,62,78].

## 4. Conclusions

In this study, the removal of fluoride from aqueous solutions was examined in a continuous fixed-bed adsorption column system using VPum and VSco. The characterizations investigations were performed using XRD, SEM, FTIR, BET, XRF, and ICP-OES equipment to reveal the mechanisms of adsorption and the suitability of the adsorbents for fluoride removal. The pH_PZC_ is 6.98 for VSco and 6.85 for VPum. The effects of process parameters such as adsorbent particle size, influent pH, and influent volumetric flow-rate on the performance of the adsorption process in a column were evaluated. The maximum removal capacity of 110 mg/kg for VPum and 22 mg/kg for VSco were achieved at a particle size of 0.075–0.425 mm and <0.075 mm, respectively, at lower solution pH (2.00) and flow rate (1.25 mL/min). The increase in adsorbent particle size, solution pH, and flow rate decreases the breakthrough and saturation time of the column bed and, consequently, lowers the amount of fluoride removal by VSco. The breakthrough and exhaustion time on VPum was high at a particle size of 0.075–0.425 mm, at lower solution pH and flow rate similar to that of VSco. Thus, in order to attain optimum performance, suitable experimental parameters are significant for the operation of the adsorption column. The Thomas and Adams–Bohart models were applied to the experimental data to estimate the breakthrough curves and to determine fixed-bed column kinetic parameters. Both the Adam–Bohart and the Thomas models could predict very well the entire region of the breakthrough curves for the fluoride-VSco and fluoride-VPum system. The results show that VPum and VSco could be used in a fixed-bed adsorption column for the removal of excess fluoride from water. The supply cost of the two adsorbents is very low; nevertheless, an overall cost analysis of the purification system is very important as it has implications for the feasibility (technical and economic) of the adsorption method. Additional testing of the adsorbents including representative samples test for possible compositional, mineralogical, and textural changes in time due to weathering, leaching test, competitive ions effects, and regeneration options is required to confirm that the defluoridation of groundwater employing volcanic rocks is a safe and sustainable method.

## Figures and Tables

**Table 1:** Material properties and experimental conditions.

Parameters	Virgin Scoria (VSco)			Virgin Pumice (VPum)		
Particle Size (mm)	<0.075	0.075–0.425	0.425–2.00	<0.075	0.075–0.425	0.425–2.00
Mass of adsorbents, m_ads_ (gm)	737.90	763.90	680.50	376.40	265.90	186.40
Bulk density, ρ_b_ (gm cm^−3^)	1.43	1.48	1.32	0.73	0.52	0.36
Particle density, ρ_s_ (gm cm^−3^)	2.37	2.33	1.94	1.61	1.31	0.64
Void volume, V_v_ (cm^3^)	203.70	187.20	164.70	281.10	311.80	222.30
Total porosity, ε	0.40	0.36	0.32	0.55	0.61	0.43
Flow rate, Q (cm^3^ min^−1^)	1.25	2.50	3.75	1.25	2.50	3.75
Empty Bed Contact Time, _EBCT_ (min)	412.00	206.00	137.33	412.00	206.00	137.33
Filter (Superficial) velocity, V_f_ (cm min^−1^)	0.02	0.05	0.07	0.02	0.05	0.07
Effective (Interstitial) velocity, V_I_ (cm min^−1^)	0.06	0.13	0.23	0.05	0.08	0.17

**Table 2:** Fixed-bed column parameters obtained for fluoride adsorption onto VPum and VSco.

**VPum.**	**H (cm)**	**C_O_** **(mg/L)**	**Q_O_** **(mL/min)**	**pH**	**Particles size** **(P_size_)** **(mm)**	**t_b_** **(min)**	**t_e_** **(min)**	**V_b_ (mL)**	**V_e_ (mL)**	**MTZ** **(cm)**	**EBCT** **(min)**	**q_tot_** **(mg)**	**q_e_** **(mg/kg)**
	10	10	1.25	2.00	<0.075	816	1623	1019.64	2033.41	4.99	412	20.28	59.6
	10	10	1.25	2.00	0.075–0.425	1206	2339	1507.50	2923.70	4.84	412	29.24	109.9
	10	10	1.25	2.00	0.425–2.00	235	1013	293.23	1265.89	7.68	412	12.67	67.9
	10	10	1.25	4.00	0.075–0.425	278	500	347.50	625	4.44	412	6.25	23.51
	10	10	1.25	6.00	0.075–0.425	135	315	168.75	393.75	5.71	412	3.94	14.81
	10	10	2.50	2.00	0.075–0.425	215	634	538.47	1585.16	6.60	206	7.93	29.8
	10	10	3.75	2.00	0.075–0.425	75	359	282.69	1346.42	7.90	137	4.49	16.89
**VSco**	**H (cm)**	**C_O_ (mg/L)**	**Q_O_** **(mL/min)**	**pH**	**Particles size** **(P_size_)(mm)**	**t_b_** **(min)**	**t_e_** **(min)**	**V_b_ (mL)**	**V_e_ (mL)**	**MTZ** **(cm)**	**EBCT** **(min)**	**q_tot_** **(mg)**	**q_e_** **(mg/kg)**
	10	10	1.25	2.00	<0.075	415	1286	518.03	1607.60	6.77	412	16.08	22
	10	10	1.25	2.00	0.075–0.425	199	760	248.99	849.80	7.38	412	9.50	12.4
	10	10	1.25	2.00	0.425–2.00	231	591	288.17	739.12	6.10	412	7.39	10.9
	10	10	1.25	4.00	<0.075	296	487	370	608.75	3.92	412	6.09	8.2
	10	10	1.25	6.00	<0.075	227	393	283.75	491.25	4.22	412	4.91	6.7
	10	10	2.50	2.00	<0.075	185	445	462.95	1113.19	5.84	206	5.57	7.5
	10	10	3.75	2.00	<0.075	69	249	256.82	931.87	7.24	137	3.10	4.2

t_b_ = breakthrough time, t_e_ = exhaustion time, V_b_ = total effluent volume at breakthrough time, V_e_ = total effluent volume at exhaustion time MTZ = Mass Transfer Zone, EBCT = Empty Bed Contact Time, q_total_ = total amount of fluoride adsorbed from the column, q_e_ = equilibrium fluoride uptake per kg of adsorbent.

**Table 3:** Thomas model parameters for fluoride adsorption onto VPum and VSco.

**VPum**	**H (cm)**	**C_o_ (mg/L)**	**Q** **(mL/min)**	**pH**	**P_size_ (mm)**	**K_T_ (L/min.mg) (×10^4^)**	**q_0(cal.)_** **(mg/kg)**	**q_e(exp.)_** **(mg/kg)**	**R^2^**
	10	10	1.25	2.00	<0.075	2.199	48.6	59.6	0.950
	10	10	1.25	2.00	0.075–0.425	1.440	109.9	109.9	0.993
	10	10	1.25	2.00	0.425–2.00	1.840	57.9	67.9	0.897
	10	10	1.25	4.00	0.075–0.425	8.289	17.83	23.51	0.977
	10	10	1.25	6.00	0.075–0.425	12.099	13.08	14.81	0.995
	10	10	2.50	2.00	0.075–0.425	3.565	55.81	29.8	0.953
	10	10	3.75	2.00	0.075–0.425	5	45.82	16.9	0.962
**VSco**	**H (cm)**	**C_o_ (mg/L)**	**Q** **(mL/min)**	**pH**	**P_size_ (mm)**	**K_T_ (L/min.mg) (×10^4^)**	**q_0(cal.)_** **(mg/kg)**	**q_e(exp.)_** **(mg/kg)**	**R^2^**
	10	10	1.25	2.00	<0.075	1.23	19	22	0.901
	10	10	1.25	2.00	0.075–0.425	2.213	13.4	12.4	0.931
	10	10	1.25	2.00	0.425–2.00	3.310	10.1	10.9	0.956
	10	10	1.25	4.00	<0.075	10.140	7.06	8.2	0.973
	10	10	1.25	6.00	<0.075	15.520	5.42	6.7	0.965
	10	10	2.50	2.00	<0.075	5.263	13.3	7.5	0.929
	10	10	3.75	2.00	<0.075	7.220	11.2	4.2	0.944

**Table 4:** Adams–Bohart model parameters for fluoride adsorption onto VPum and VSco.

**VPum**	**H (cm)**	**C_o_ (mg/L)**	**Q** **(mL/min)**	**pH**	**P_size_** **(mm)**	**K_AB_ (L/min.mg) (×10^4^)**	**N_0_** **(mg/L)**	**R^2^**
	10	10	1.25	2.00	<0.075	2.187	35.55	0.950
	10	10	1.25	2.00	0.075–0.425	1.439	56.85	0.993
	10	10	1.25	2.00	0.425–2.00	1.741	20.77	0.911
	10	10	1.25	4.00	0.075–0.425	8. 289	9.22	0.995
	10	10	1.25	6.00	0.075–0.425	12. 099	6.76	0.953
	10	10	2.50	2.00	0.075–0.425	3.565	29.68	0. 962
	10	10	3.75	2.00	0.075–0.425	5.000	22.74	0.995
**VSco**	**H(cm)**	**C_o_ (mg/L)**	**Q** **(mL/min)**	**pH**	**P_size_** **(mm)**	**K_AB_ (L/min.mg) (×10^4^)**	**N_o_** **(mg/L)**	**R^2^**
	10	10	1.25	2.00	<0.075	1.233	27.27	0.886
	10	10	1.25	2.00	0.075–0.425	2.213	17.68	0.886
	10	10	1.25	2.00	0.425–2.00	3.310	13.41	0.956
	10	10	1.25	4.00	< 0.075	10.145	10.13	0.969
	10	10	1.25	6.00	< 0.075	15.518	7.77	0.980
	10	10	2.50	2.00	< 0.075	5.263	19.72	0.929
	10	10	3.75	2.00	< 0.075	7.221	15.36	0.944

**Table 5:** Comparison of other adsorbents with VPum and VSco.

Adsorbents	Surface Area (m^2^g^−1^)	Bed Depth (cm)	Fluoride in (mgL^−1^)	Adsorption Capacity (mg F^-^ g^−1^)	Adsorption Capacity per Surface Area (mg.m^−2^)	References
Cement paste	NA*	20	15	0.149	-	[61]
aluminum modified iron oxide	NA	10.5	4	0.139	-	[62]
Acid-treated bentonite (GHB)	24.5	28	2.85	0.169	0.0069	[63]
MnO_2_-coated Tamarind Fruit Shell	NA	6	2	0.883	-	[64]
kanuma mud	144.01	10	20	1.560	0.0108	[49]
Activated alumina (Grade OA-25)	250	10	5	0.74	0.0029	[65]
VPum	3.5	10	10	0.110	0.0314	This study
VSco	2.49	10	10	0.022	0.0088	This study

NA*: Not available.

## Data Availability

The data used in this study can be available from the authors on reasonable request.

## Supplementary Materials

Table S1: Elemental composition and oxides content of VPum and VSco, Figure S1: Experimental (exp.) and simulated (cal.; Thomas model) breakthrough curves of fluoride at different particle sizes for (a) VPum and (b) VSco (pH 2.00; C_O_: 10 mg/L; Q_O_: 1.25 mL/min; bed depth 10 cm), Figure S2: Experimental and simulated (Thomas model) breakthrough curves of fluoride at different pH for (a) VPum: 0.075–0.425 mm (b) VSco: <0.075 mm (CO: 10 mg/L; QO: 1.25 mL/min; bed depth 10 cm), Figure S3: Experimental and simulated (Thomas model) breakthrough curves of fluoride at different influent flow rate for (a) VPum: 0.075–0.425 mm and (b) VSco: <0.075 mm (pH 2.00; C_O_: 10 mg/L; bed depth 10 cm), Figure S4: Experimental (exp.) and simulated (cal.; Adams–Bohart model) breakthrough curves of fluoride at different particle sizes for (a) VPum and (b) VSco (pH 2.00; C_O_: 10 mg/L; Q_O_: 1.25 mL/min; bed depth 10 cm), Figure S5: Experimental and simulated (Adams–Bohart model) breakthrough curves of fluoride at different pH for (a) VPum: 0.075–0.425 mm and (b) VSco: <0.075 mm (C_O_: 10 mg/L; Q_O_: 1.25 mL/min; bed depth 10 cm), Figure S6: Experimental and simulated (Adams–Bohart model) breakthrough curves of fluoride at different flow rate for (a) VPum: 0.075–0.425 mm and (b) VSco: <0.075 mm (pH 2.00; C_O_: 10 mg/L; bed depth 10 cm).

## Sample Availability

Samples of the adsorbents (VPum and VSco) are available from the authors.
