# Supplementary material for: Volcanic Rock Materials for Defluoridation of Water in Fixed-Bed Column Systems
Source: Molecules. 2021 Feb 12;26(4):977. doi: 10.3390/molecules26040977 (PMC7918344; doi:10.3390/molecules26040977)
Supplement: Supplementary file 1 [file molecules-26-00977-s001.pdf]

molecules

Supplementary Materials

Table S1. Elemental composition and oxide content of VPum and VSco.

Elements	VPum % (wt)	VSco % (wt)	Oxides	VPum % (wt)	VSco % (wt)
Si	27.1	18.3	SiO_2	68.6	47.4
Al	5.3	10.3	Al_2O_3	8.9	21.6
Fe	3.4	7.8	Fe_2O_3	4.9	8.9
K	3.8	0.4	K_2O	5.5	0.5
Ca	0.3	6.4	CaO	1.8	12.4
Na	1.2	2.2	Na_2O	4.1	3.0
Mg	0.1	2.8	MgO	0.2	3.3
Zn	<0.1	<0.1	TiO_2	0.3	1.2
Mn	<0.1	0.1	Others	5.7	1.2
Cr	<0.1	<0.1			
Cu	<0.1	<0.1			
Co	<0.1	<0.1			
Cd	<0.1	<0.1			
Ni	<0.1	<0.1			
Pb	<0.1	<0.1			
As	<0.1	<0.1			

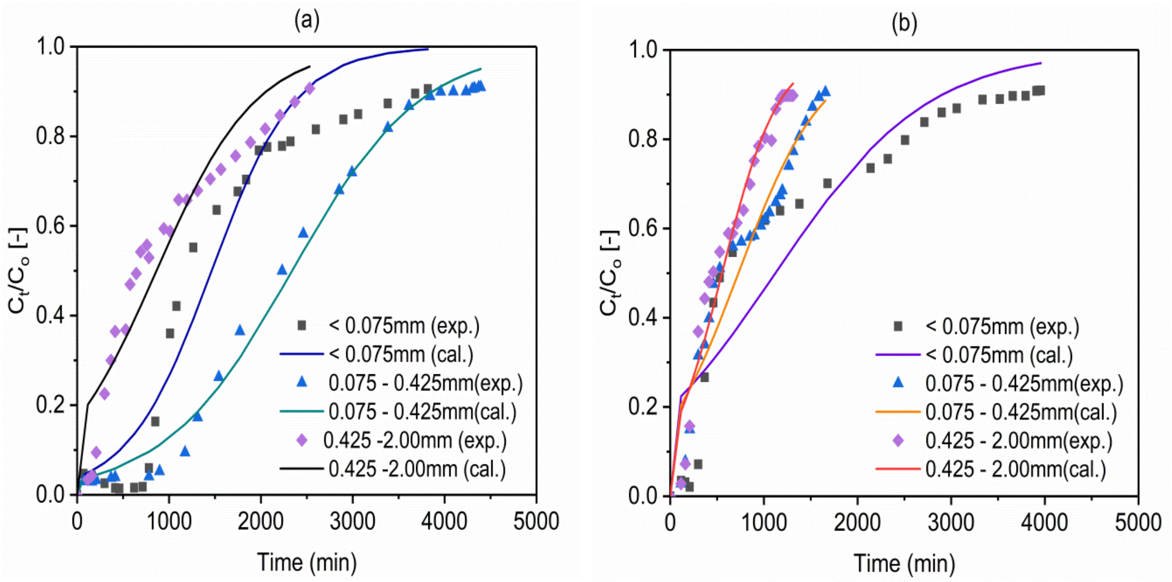

Figure S1. Experimental (exp.) and simulated (cal.; Thomas model) breakthrough curves of fluoride at different particle sizes for (a) VPum and (b) VSco (pH 2.00; C_o: 10 mg/L; Q_o: 1.25 mL/min; bed depth 10 cm).

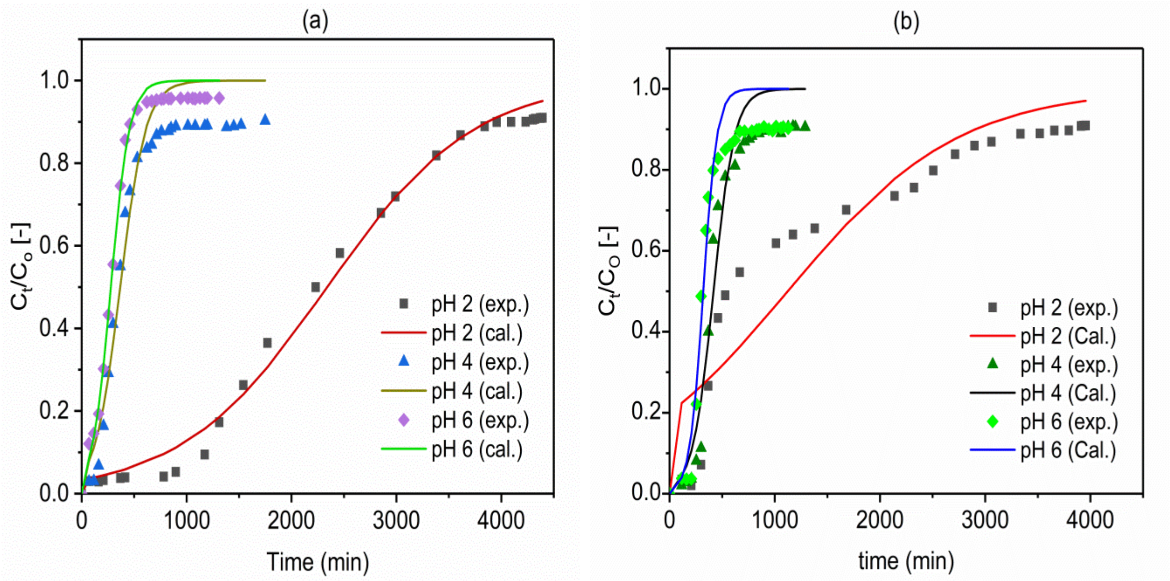

Figure S2. Experimental and simulated (Thomas model) breakthrough curves of fluoride at different pH for (a) VPum: 0.075 – 0.425 mm (b) VSco: < 0.075 mm (Co: 10 mg/L; Qo: 1.25 mL/min; bed depth 10 cm).

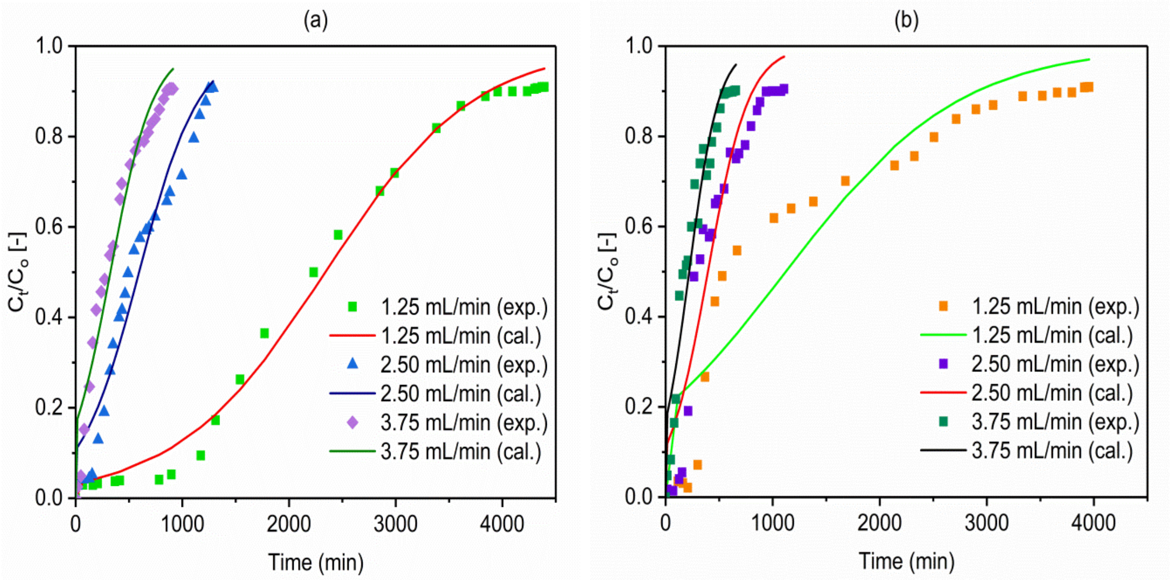

Figure S3. Experimental and simulated (Thomas model) breakthrough curves of fluoride at different influent flow rate for (a) VPum: 0.075 – 0.425 mm and (b) VSco: < 0.075 mm (pH 2.00; Co: 10 mg/L; bed depth 10 cm).

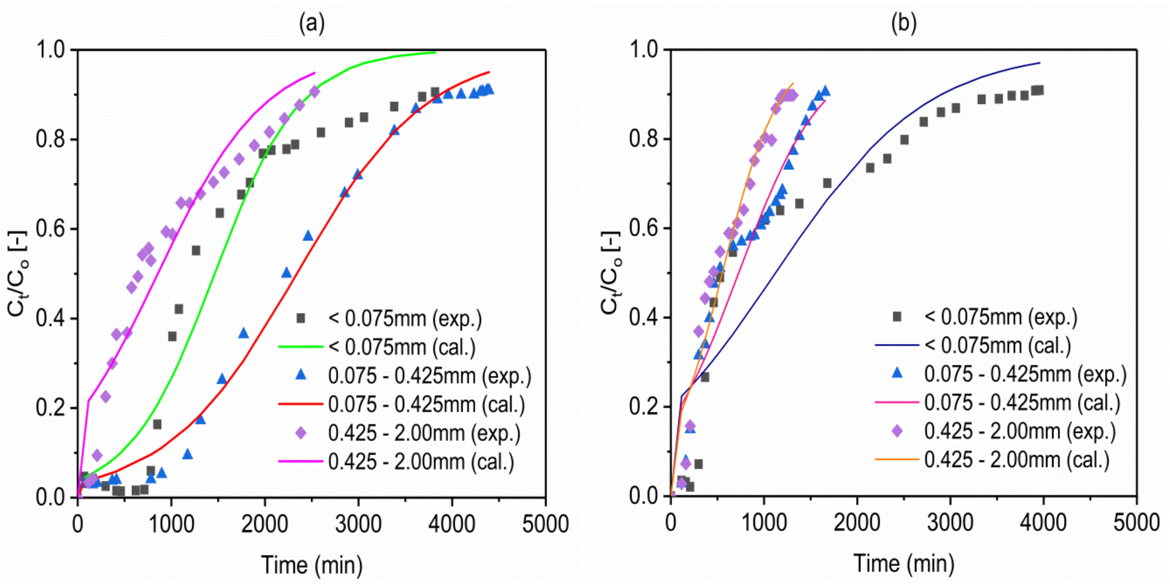

Figure S4. Experimental (exp.) and simulated (cal.; Adams-Bohart model) breakthrough curves of fluoride at different particle sizes for (a) VPum and (b) VSco (pH 2; Co: 10 mg/L; Qo: 1.25 mL/min; bed depth 10 cm).

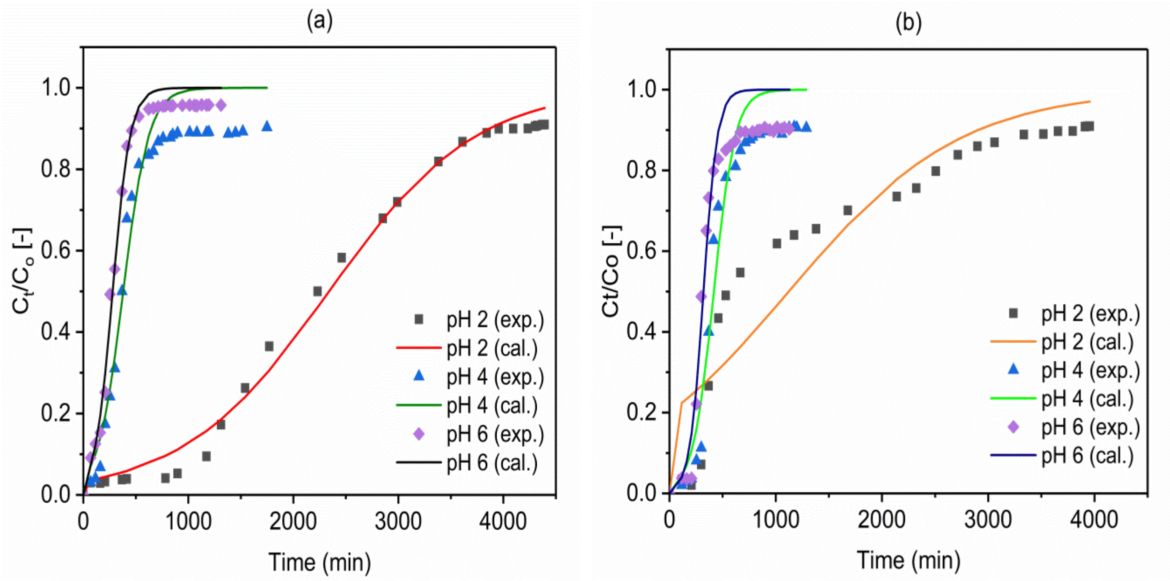

Figure S5. Experimental and simulated (Adams-Bohart model) breakthrough curves of fluoride at different pH for (a) VPum: 0.075 – 0.425 mm and (b) VSco: < 0.075 mm (Co: 10 mg/L; Qo: 1.25 mL/min; bed depth 10 cm).

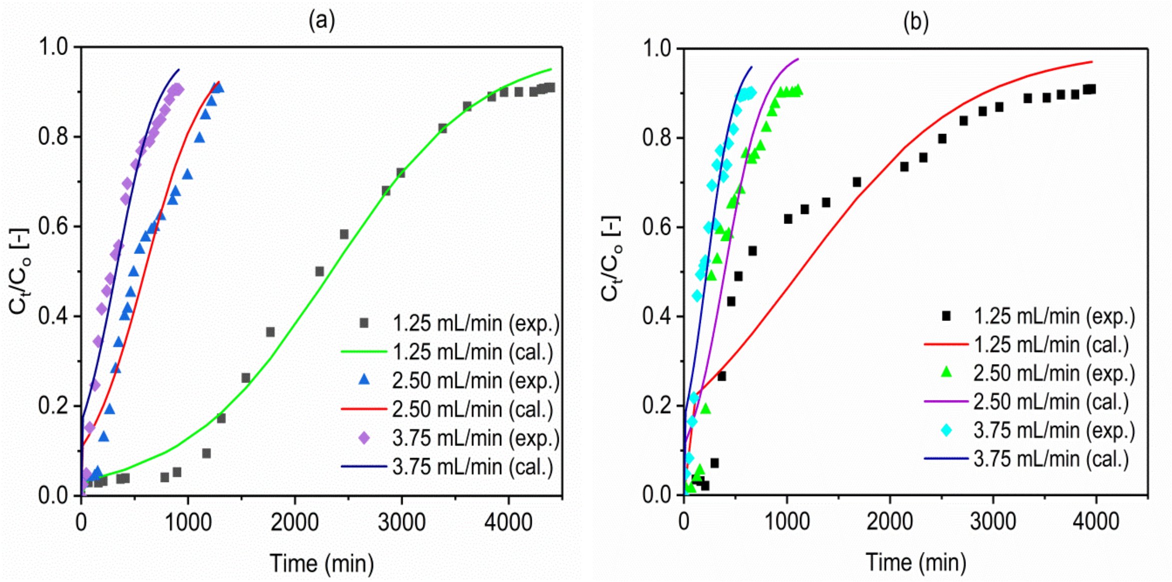

Figure S6. Experimental and simulated (Adams-Bohart model) breakthrough curves of fluoride at different flow rate for (a) VPum: 0.075 – 0.425 mm and (b) VSco: < 0.075 mm (pH 2.00; C_O: 10 mg/L; bed depth 10 cm).
